# Dual function of angiogenin-4 inducing intestinal stem cells and apoptosis

**DOI:** 10.3389/fcell.2023.1181145

**Published:** 2023-11-02

**Authors:** Hirohito Abo, Mst. Farzana Sultana, Hiroto Kawashima

**Affiliations:** ^1^ Laboratory of Microbiology and Immunology, Graduate School of Pharmaceutical Sciences, Chiba University, Chiba, Japan; ^2^ Department of Pharmacy, Jashore University of Science and Technology, Jashore, Bangladesh

**Keywords:** stem cells, antimicrobial peptide (AMP), intestinal epithelium, Wnt signalling, cell differentiation

## Abstract

The intestinal epithelium is the first line of host defense, and its homeostasis is dependent on soluble factors that comprise the crypt niche. Antimicrobial proteins are one of the mediators to maintain gut homeostasis. Angiogenin-4 (Ang4) is a member of the ribonuclease A superfamily and plays a pivotal role in antimicrobial activity against gut microbiota. However, the functions of Ang4 within the intestinal crypt niche, particularly its involvement in the development of intestinal epithelial cells (IECs), remain unknown. Here, we demonstrate that Ang4 plays a significant role in maintaining Lgr5^+^ intestinal stem cells (ISCs) and induces apoptosis of IECs in a concentration-dependent manner. We revealed that Ang4 is highly expressed by Paneth cells in the small intestine, as well as regenerating islet-derived family member-4 (Reg4) expressing goblet cells in the colon, and both cell subsets highly contribute to ISC maintenance. Functional analysis using intestinal organoids revealed that Ang4 induces Wnt and Notch signaling, increases Lgr5^+^ stem cell expansion, and promotes organoid growth. Furthermore, high concentrations of Ang4 induced apoptosis in the IEC cell line and organoids. Collectively, we propose that Ang4 is a dual functional protein and is a novel member of the crypt niche factor that promotes the expansion of ISCs and induces apoptosis.

## 1 Introduction

Intestinal epithelial cells (IECs) maintain gut homeostasis by generating a diverse array of antimicrobial peptides (AMPs), immune-modulating cytokines and chemokines in response to commensal and/or pathogens. In response to bacterial invasion or epithelial damage, IECs rapidly enhance the expression of AMPs to limit bacterial encroachment. In addition, AMPs shape the composition of gut microbiota and help maintain intestinal homeostasis (Muniz et al., 2012). Recent studies have highlighted that reduced AMP expression in the gut is responsible for intestinal diseases (Mukherjee and Hooper, 2015). For example, individuals with XBP1 mutations have a higher risk of inflammatory bowel disease owing to Paneth cell abnormalities and diminished AMP secretion (Kaser et al., 2008). Therefore, comprehensive research on the functions of AMPs can lead the better understanding of gut homeostasis and treatment of intestinal diseases.

Leucine-rich repeat-containing G protein-coupled receptor 5 (Lgr5)-expressing intestinal stem cells (ISCs) differentiate into all epithelial lineages including Paneth cells, tuft cells, enteroendocrine cells, goblet cells, and enterocytes along the crypt-villus axis. These differentiation steps are highly influenced by the cell-cell networks in the crypt niche. In the small intestine, Paneth cells play an essential role in the maintenance of ISCs via the production of stem cell supporting factors including EGF, Wnt3, and Dll4, as evidenced by the removal of Paneth cells *in vivo*, resulting in the concomitant loss of Lgr5^+^ ISCs (Sato et al., 2011). Furthermore, previous report demonstrated that enteroendocrine and tuft cells occupy the position between Lgr5^+^ stem cells and provide essential signals for stem cell maintenance after deletion of Paneth cells (van Es et al., 2019). In addition, regenerating islet-derived family member-4 (Reg4)^+^ deep crypt secretory (DCS) cells, which is a subpopulation of goblet cells, show properties similar to Paneth cells and fulfill similar functions in the colon (Sasaki et al., 2016). Multiple signaling pathways, including Notch, Wnt, Hippo, and Bmp, coordinate proliferation and differentiation to maintain continuous production of the diverse cell types necessary to preserve stem cell pools (Santos et al., 2018). Especially, canonical Wnt signaling is a well-established component of homeostatic self-renewal and proliferation of ISCs (Storm et al., 2016; Yan et al., 2017).

Angiogenin-4 (Ang4) is a member of the ribonuclease A superfamily and acts as a multifunctional protein involved in angiogenesis, tumorigenesis, neuroprotection, antibacterial activity, and innate immunity (Hooper et al., 2003; Sheng and Xu, 2016). In the intestinal tract, Ang4 functions as an endogenous antimicrobial protein and is secreted into the gut lumen along with other secretory components, such as lysozyme, Reg3β, and Reg3γ (Hooper et al., 2003; Burger et al., 2012). It has been confirmed that Ang4 is expressed in the intestine by Paneth cells and goblet cells following microbial induction, as evidenced by a significant reduction in Ang4 expression in germ-free mice (Hooper et al., 2003; Forman et al., 2012). In addition, Ang4 functions as a regulatory factor for gut microbiota (Sultana et al., 2022). Although the fundamental role of Ang4 as an antimicrobial peptide and its effect on gut microbiota is well established, comparative details of Ang4 expression and its function in IECs, especially IEC differentiation, remain to be elucidated.

In this study, we explored the dual role of Ang4 on IECs. Using single-cell RNA sequencing data, we revealed that Ang4 was most highly expressed by Paneth cells in the small intestine and co-expressed with Reg4^+^ DCS cells in the large intestine, indicating the potential of Ang4 as a crypt niche factor to control Lgr5^+^ stem cell maintenance and IEC differentiation. Using *in vitro* organoid culture system, we demonstrated that Ang4 enhanced organoid growth and increased expression of ISC signature genes through the upregulation of Wnt and Notch signaling as evidenced by the increased expression of target genes. In addition, administration of Ang4 into the mice increased ISCs. In contrast, treatment of organoids with Ang4 at high concentrations induced apoptosis against IECs. Collectively, our findings suggest that Ang4 is a bi-functional protein inducing the expansion of Lgr5^+^ stem cells and IEC apoptosis.

## 2 Materials and methods

### 2.1 Mice

Female C57BL/6J mice were obtained from Charles River Laboratories Japan, and maintained in the Chiba University animal facility in accordance with the guidelines of the Animal Care and Use Committee of Chiba University. Age- and sex- matched mice were used for all experiments.

### 2.2 Analysis of single-cell RNA sequencing data

Processed single-cell RNA sequencing data were obtained from Gene Expression Omnibus (accession number: GSE148693) and analyzed using the Seurat package (32). The expression matrix contained filtered cells that had unique feature counts over 2,500 or less than 200 and discarded cells that contained >5% mitochondrial unique molecular identifier counts. After quality control of the dataset, we employed normalization by a scale factor of 10,000 following dimensionality reduction using principal component analysis. The principal components were subjected as an input for clustering of cells by graph-based clustering using FindNeighbors and FindClusters. The genes that were specifically expressed in each cluster were identified using the FindAllMarker function.

### 2.3 Generation and purification of recombinant Ang4

Generation and purification of recombinant Ang4 were performed as previously described (Sultana et al., 2022). Briefly, synthetic His-tagged Ang4 containing the sequences of restriction enzymes *Nco*I and *Hin*dIII with codon optimization for efficient gene expression in *Brevibacillus choshinensis* was cloned into vector pNCMO2, followed by transformation into *E. coli* DH5α. After confirming the sequence of the target DNA of Ang4, the plasmid DNA was transformed into *Brevibacillus* competent cells for protein expression. Then, *Brevibacillus* pNCMO2-Ang4 transformants were cultured in a 2SYNm medium at 30°C for 64 h. The supernatant was then subjected to 70% ammonium sulfate precipitation, followed by purification using His60 Ni Superflow™ resin (635,659; TAKARA BIO. Inc., Shiga, Japan) and HiTrap cation exchange chromatography (29051324; Cytiva, Marlborough, MA, United States). The purity≥95% and concentration of Ang4 proteins were examined by SDS-PAGE and BCA protein assay kits, respectively.

### 2.4 Isolation of crypt cells and organoid cultures

Crypt cell isolation and organoid culture were performed as previously described (Sato et al., 2009). For the isolation of crypts, the small and large intestine was longitudinally cut into 5 mm pieces and washed with cold PBS. Then, a gentle cell dissociation reagent (100–0485; Stem Cell Technologies, Vancouver, Canada) was added and kept at room temperature for 1 h with continuous shaking (30 rpm). After that, 5% FBS in PBS was added to tissues and pipetted 10 times to release crypts from tissues. Then, the crypt-containing fraction was enriched by centrifuging twice at 4°C, 300 × *g* for 3 min. After collecting the crypts in organoid medium (06,005; Stem Cell Technologies), crypts were embedded in the Matrigel (200 crypts/well, 356,234; Corning Inc., Corning, NY, United States) and polymerized by incubation at 37°C for 15 min. After Matrigel polymerization, culture media was added and refreshed every 2 days at 37°C.

### 2.5 Stimulation of Ang4 with organoids

Organoids generated from the small and large intestine were collected in cold 5% FBS in PBS and washed by centrifugation at 4°C, 300 × *g* for 3 min. Then, organoids were pipetted 50 times and washed, followed by incubation with Matrigel at 37°C for 15 min for polymerization. Subsequently, 150 µL of Ang4 protein (5 μg/mL or 25 μg/mL) in an organoid medium was added to each well of a 48-well plate and incubated at 37°C for 4 days. Organoid measurements were performed as previously described (Lindemans et al., 2015). When passaging organoids, we first separated them into individual crypts by pipetting, and then cultured 200 distinct crypts per well. For size evaluation, the area of horizontal cross sections of the organoid was measured. Cross section perimeters required for area measurement was defined by automatically determination using the Analyze Particle function of the ImageJ software, as automated measurements allowed for unbiased analysis of increased numbers of organoids. After culture for 4 days, total matured organoid numbers were counted in each well, and organoid efficiency was calculated as the ratio of total crypt number of initially added to the well. Organoids that have new crypt formation are counted as budding organoid, defined as three or more buddings, and determined ratio of total crypt number after 4 days culture. To examine the dose titration of Ang4 in the switch of cell proliferation and apoptosis, organoids were cultured at the concentrations of 1, 5, 10, 25, and 50 μg/mL for 4 days, and then organoid growth and apoptosis were analyzed using flow cytometry (CytoFLEX; Beckman Coulter, Brea, CA, United States). For the treatment of Wnt signaling inhibitor, organoids were treated with iCRT3 (HY-103705, MedChemExpress) at the concentration of 20 μM and cultured for 4 days. For stimulation of organoids with Reg3γ, organoids were treated with recombinant Reg3γ (8189-RG-050, R&D systems, MN, United States) at the concentration of 30 μg/mL for 4 days. After 4 days culture, budding, organoid efficiency, and area of cross section were measured.

### 2.6 RNA isolation from organoids and qPCR

Total RNA from Ang4 stimulated organoids was isolated using NucleoSpin RNA (740,955.50; TAKARA BIO. Inc). We isolated total RNA of organoids after 4 days of stimulation, since organoids were mostly matured from day 4 to day 5 in this study. The cDNA was generated using PrimeScript RT Master Mix (RP036A; TAKARA BIO. Inc). Following this, qPCR was performed using SsoAdvanced Universal SYBR Green Supermix (1725270; Bio-Rad, Hercules, CA, United States) on a CFX96 Touch Real-Time PCR Detection system (Bio-Rad), and gene expression was normalized to *Actb*. The oligonucleotide primers used for quantification of gene expression were as follows:

*Lgr5* (F, 5′-GTG​GAC​TGC​TCG​GAC​CTG-3′; R, 5′-GCT​GAC​TGA​TGT​TGT​TCA​TAC​TGA​G-3′) *Ascl2* (F, 5′-GTT​AGG​GGG​CTA​CTG​AGC​AT-3′; R, 5′-GTC​AGC​ACT​TGG​CAT​TTG​GT-3′) *Smoc2* (F, 5′-CCC​AAG​CTC​CCC​TCA​GAA​G-3′; R, 5′-GCC​ACA​CAC​CTG​GAC​ACA​T-3′) *Olfm4* (F, 5′-CTG​CTC​CTG​GAA​GCT​GTA​GT-3′; R, 5′-ACC​TCC​TTG​GCC​ATA​GCG​AA-3′) *Lyz1* (F, 5′-GGA​ATG​GAT​GGC​TAC​CGT​GG -3′; R, 5′-CAT​GCC​ACC​CAT​GCT​CGA​AT-3′) *Defa24* (F, 5′-CAC​TGA​GCT​GCT​ACT​CAC​CA-3′; R, 5′-GCA​TAC​CAG​ATC​TCT​CAA​TGA​TTC​C-3′) *Agr2* (F, 5′-CGA​ATG​CCC​ACA​CAG​TCA​AG -3′; R, 5′-CGT​CAG​GGA​TGG​GTC​TAC​AAA -3′) *Muc2* (F, 5′-TGA​CTG​CCG​AGA​CTC​CTA​CA-3′; R, 5′-CTG​TAG​TGT​GGG​GTG​CTG​AC-3′) *Apoa1* (F, 5′-GAG​GTC​ACC​CAC​ACC​CTT​CA -3′; R, 5′-AGT​TTT​CCA​GGA​GAT​TCA​GGT​TCA-3′) *Trpm5* (F, 5′-GGA​CGT​GGA​ATG​GAA​GTC​CTG -3′; R, 5′-AGT​GTC​AGC​CTA​CCC​TCC​TC-3′) *Chga* (F, 5′-GCA​GAG​GAC​CAG​GAG​CTA​GA -3′; R, 5′-CAG​GGG​CTG​AGA​ACA​AGA​GA -3′) *Axin2* (F, 5′-GCT​CCA​GAA​GAT​CAC​AAA​GAG​C-3′; R, 5′-AGC​TTT​GAG​CCT​TCA​GCA​TC-3′) *Myc* (F, 5′-CGC​GAT​CAG​CTC​TCC​TGA​AA-3′; R, 5′-GCT​GTA​CGG​AGT​CGT​AGT​CG-3′) *Sox9* (F, 5′-TGA​AGA​ACG​GAC​AAG​CGG​AG-3′; R, 5′-CAG​CTT​GCA​CGT​CGG​TTT​TG-3′) *Ephb2* (F, 5′-TAC​AAC​GCC​ACG​GCC​ATA​AA-3′; R, 5′-CCA​ACG​ATG​AGG​GGT​AGC​TT-3′) *Hes1* (F, 5′-CCA​GCC​AGT​GTC​AAC​ACG​A-3′; R, 5′-AAT​GCC​GGG​AGC​TAT​CTT​TCT-3′) *Yap1* (F, 5′-CGG​CAG​TCC​TCC​TTT​GAG​AT-3′; R, 5′-TTC​AGT​TGC​GAA​AGC​ATG​GC-3′) *Cyr61* (F, 5′-CAG​TGC​TGT​GAA​GAG​TGG​GT-3′; R, 5′-GCG​TGC​AGA​GGG​TTG​AAA​AG-3′) *Actb* (F, 5-CAT​CCG​TAA​AGA​CCT​CTA​TGC​CAA​C -3′; R, 5-ATG​GAG​CCA​CCG​ATC​CAC​A -3′).

### 2.7 RNA sequencing analysis

RNA isolation from organoids was performed as described in Section 2.6. Total RNA was purified from total RNA using poly-T oligo-attached magnetic beads. After fragmentation, the first strand cDNA was synthesized using random hexamer primers followed by the second strand cDNA synthesis. The library was prepared after end repair, A-tailing, adapter ligation, size selection, amplification, and purification. The library was checked with Qubit and real-time PCR for quantification and bioanalyzer for size distribution detection. Quantified libraries were sequenced on Illumina platforms. Law data were processed using Trimmomatic, HISAT2, and featureCount software. Differential gene expression analysis was performed using DESeq2. Gene set enrichment analysis (GSEA) was performed with 1000 permutations (https://www.gsea-msigdb.org/gsea/index.jsp). Mouse hallmark gene sets and Hippo signaling gene set were available in the GSEA Molecular Signature Database. Gene sets with a FDR of <0.25 were considered significant.

### 2.8 Treatment of mice with recombinant Ang4

For recombinant Ang4 treatment, mice were intraperitoneally injected on days 0, 2, 4, and 6 with 2 µg of Ang4 and analyzed the results on day 7. To examine the dose titration of Ang4 in the switch of cell proliferation, mice were injected with Ang4 at a dose of 2, 5, 10, and 25 μg every 2 days, and results were analyzed on day 7.

### 2.9 Immunofluorescence staining

For immunofluorescence staining, ileum and large intestinal tissues were fixed in 10% formalin, embedded in paraffin, and cut into 10 µm slices. After deparaffinization, permeabilization of tissue sections was performed with 0.3% TritonX-100 and blocked in 3% BSA for 1 h at room temperature prior to incubation overnight with anti-Ki-67 antibody (9029; Cell Signaling Technology, Danvers, MA, United States; 1:500 dilution) or anti-Olfm4 antibody (39,141; Cell Signaling Technology; 1:500 dilution) or anti-β-catenin antibody (8480; Cell Signaling Technology; 1:500 dilution) at 4°C. Alexa Fluor 594-conjugated anti-rabbit IgG secondary antibody (AS039, ABclonal, 1:1000 dilution) was added and incubated for 1 h at room temperature. For staining of organoid, small intestinal organoids were fixed by 4% PFA and embedded by OCT. Frozen sections cut in 7 μm-thickness were stained followed by describing above. Images of the sections were obtained using a fluorescence microscope BZ-800 (KEYENCE, Osaka, Japan). For counting of target cells (positive for Ki-67, Olfm4, Lysozyme, and β-catenin), we counted all crypt cells that are completely visible bottom to the villus. For all crypts meeting these conditions, we counted the target cells and calculated the proportion for each crypt. This was done for all the crypts present in a single image, and their averages were taken as a single data point.

### 2.10 Cell cycle and apoptosis assay in MC38 cell line

MC38 cells were cultured in DMEM in the presence of 10% FBS, 100 μg/mL streptomycin, and 100 IU penicillin. MC38 cells were collected with trypsin and washed with PBS, followed by suspension of the pellet in 1 mL DMEM. An appropriate number of cells was then mixed with different concentrations of Ang4 protein, plated in a 96-well plate, and incubated at 37°C for 24 h. For cell cycle analysis, BrdU was added at the concentration of 10 μM and incubated for 1 h before cell collection. After completion of incubation, cells were collected with trypsin in a 96-well plate and centrifuged at 4°C, 1500 rpm for 3 min. Cell fixation was performed by adding 70% cold ethanol and incubating for 1 h on ice, followed by washing three times with PBS. Next, cells were treated with 2N HCl at 25°C for 30 min and neutralized with 0.1 M Ba_2_B_4_O_7_. After washing twice with PBS, cells were stained with an anti-BrdU antibody (364,107, BioLegend) suspended in 0.1% Triton X-100/0.1% BSA/PBS. Cells were washed twice with PBS, and then 200 µL of a mixture containing PI (1 μg/mL), RNase (40 μg/mL), and 0.05% Triton X-100 was added to each well and incubated at 25°C for 30 min. The cell cycle was analyzed using flow cytometry. For the detection of apoptosis, 100 µL annexin V buffer (422,201, BioLegend, San Diego, CA, United States) was added to each well containing MC38 cells and washed by centrifugation at 4°C, 1500 rpm for 3 min. Then, 30 µL of annexin V (640,919, BioLegend) and PI solution was added to each well and incubated at room temperature for 15 min. Then, 150 µL of annexin V was added to each tube and analyzed using flow cytometry (CytoFLEX; Beckman Coulter, Brea, CA, United States).

### 2.11 Flow cytometry for detection of cell cycle and apoptosis from organoids

For cell cycle analysis, BrdU was added at the concentration of 10 μM and incubated 2 h before collecting organoids. Organoids stimulated with Ang4 were collected with 5% FBS in PBS and washed by centrifugation at 4°C, 300 × *g* for 3 min. Next, 500 µL of TrypLE Express (12604013, Gibco, Waltham, MA, United States) was added to organoids and incubated at 37°C for 10 min. After incubation, organoids were pipetted, passed through a 70 μm mesh, and washed with 5% FBS in PBS. The organoids were suspended with 5% FBS in PBS and added to a 96-well plate. For cell cycle analysis, cells were prepared as described in Section 2.10. For apoptosis detection, cells were washed with 200 µL of annexin V buffer (422,201, BioLegend). Then, 50 µL of solution mixture containing APC conjugated annexin V (640,919, BioLegend) and propidium iodide (PI) in annexin V buffer was added to each well and incubated at 4°C for 15 min. Then, 150 µL of annexin V buffer was added to each well and analyzed using flow cytometry (Beckman, CytoFLEX).

### 2.12 Statistical analysis

The presented data were pooled from two or three independent experiments. All statistical analyses were performed using GraphPad Prism software version 9. ANOVA followed by Tukey’s multiple comparison test or non-parametric Mann-Whitney test was used to determine significance (*p < 0.05, **p < 0.01, ***p < 0.001).

## 3 Results

### 3.1 Analysis of Ang4 expression within IEC subtypes

**FIGURE 1:**
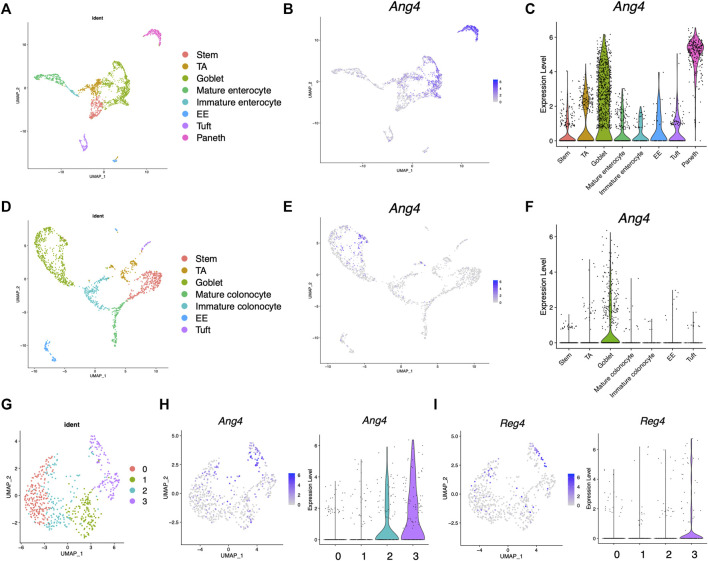
Expression profile of Ang4 in the small and large intestine. **(A)** UMAP projection of single-cell RNA sequencing profile from healthy mouse ileum (data from GSE14809). **(B)** Feature plots highlighting expression of Ang4 in ileum. **(C)** Violin plots of Ang4 gene expression between epithelial cell subsets in ileum. **(D)** UMAP projection of single-cell RNA sequencing profile from healthy mice colon (data are from GSE14809). **(E)** Feature plots highlighting expression of Ang4 in colon. **(F)** Violin plots of Ang4 gene expression between epithelial cell subsets in colon. **(G)** UMAP plot of four clusters from re-clustered colonic goblet cells. **(H)** Feature plot and violin plot of Ang4 from four clusters shown in **(G)**. **(I)** Feature plot and violin plot of Reg4 from four clusters shown in **(G)**.

Previous research has revealed that Ang4 is expressed by Paneth cells and goblet cells (Hooper et al., 2003; Forman et al., 2012). However, the expression of Ang4 among IEC subtype is not well defined. To analyze the expression of Ang4 in detail, we first analyzed single-cell RNA sequencing data deposited in a public database (Gu et al., 2022). In this analysis, cell subsets were identified using lineage-specific markers of stem cells, goblet cells, enterocytes, colonocytes, Paneth cells, tuft cells, and enteroendocrine cells (Supplementary Figures S1A, B, Supplementary Figure S2, and Supplementary Figure S3) (Haber et al., 2017). In the small intestine, Ang4 was remarkably expressed in goblet cells identified as *Muc2* and Paneth cells identified as *Lyz1*, with the latter showing higher expression of Ang4 (Figures 1A–C). In colon tissue, which lacks Paneth cells, Ang4 was observed to be highly expressed in goblet cells (Figures 1D–F). Previous studies have revealed that goblet cells have distinct clusters with different functions (Nystrom et al., 2021). Consistent with this finding, further investigations indicated four distinct clusters within the goblet cells (Figure 1G and Supplementary Figure S4). Ang4 expression was mainly detected in clusters 2 and 3, with higher expression of Ang4 in the latter cluster (Figure 1H). Furthermore, Reg4-expressing DCS cells, which is a subpopulations of goblet cells, function as a crypt niche for the maintenance of Lgr5^+^ ISCs and the colon equivalent of Paneth cells (Sasaki et al., 2016). Based on this finding, we analyzed the expression of Reg4 in the dataset. As expected, Reg4 expression was highly detected in cluster 3, which was co-expressed with Ang4 (Figure 1I). These findings reveal that Ang4 is expressed on cell subsets which have essential role on ISC maintenance, indicating that Ang4 is a possible regulatory factor that affects Lgr5^+^ ISCs and maintains the crypt niche.

### 3.2 Ang4 increases organoid growth and expression of ISC signature genes

**FIGURE 2:**
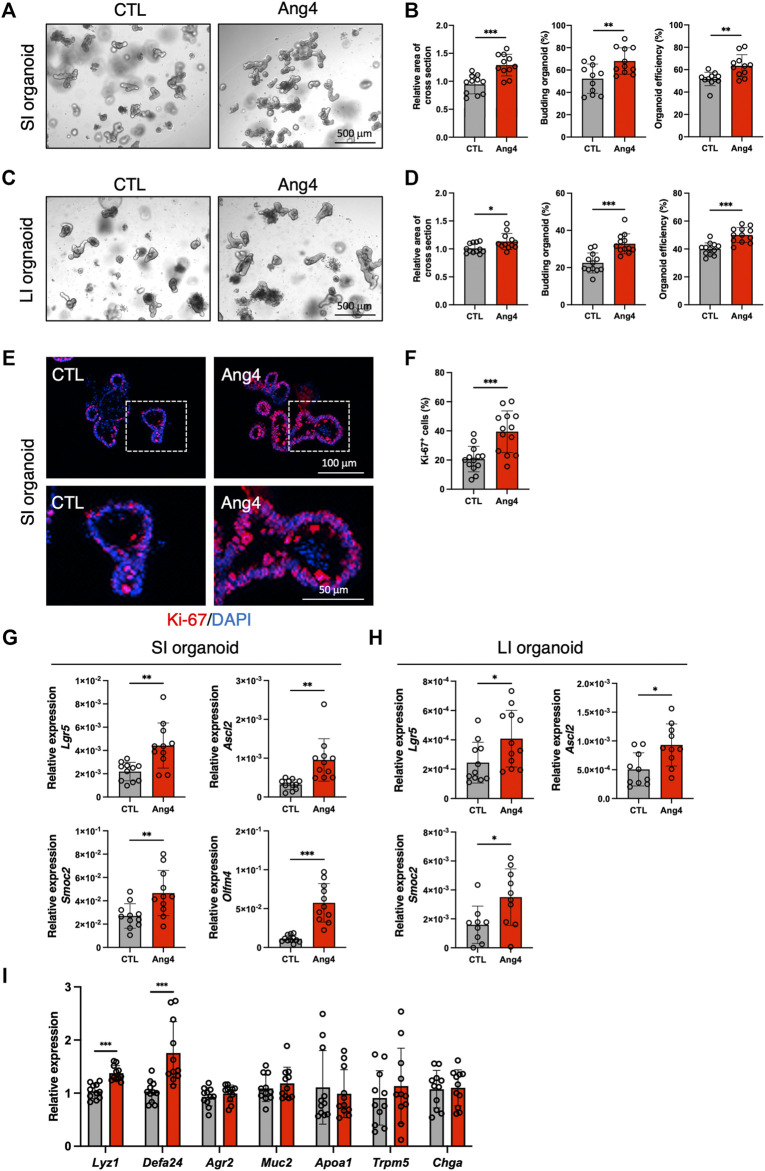
Ang4 increases organoid growth with upregulation of ISCs signature genes. **(A)** Representative pictures of small intestinal organoids cultured with or without Ang4 at a concentration of 5 μg/mL. **(B)** Area of a cross section, budding of organoids, and organoid efficiency shown in **(A)** (n = 11). **(C)** Representative pictures of colonic organoids cultured with or without Ang4 at a concentration of 5 μg/mL. **(D)** Area of a cross section, budding of organoids, and organoid efficiency shown in **(C)** (n = 12). **(E)** Representative images of Ki-67 staining in organoid cultured with or without Ang4. top panel; low magnification, bottom panel; high magnification. **(F)** Quantification of Ki-67^+^ cells shown in **(E)**. **(G)** Expression of ISC signature genes in small intestinal organoids analyzed by qPCR (n = 11). **(H)** Expression of ISC signature genes in colonic organoids analyzed by qPCR (n = 9–12). **(I)** Expression of IEC subtype specific genes in small intestinal organoids treated with or without Ang4 (n = 11). Data are pooled from three independent experiments and are presented as the mean ± SD; *p < 0.05, **p < 0.01, ***p < 0.001 via non-parametric Mann-Whitney test.

Analysis of single-cell RNA sequencing data indicated interaction between Ang4 and ISCs. Recently, three-dimensional organoids that mimic the intestinal epithelial structure have been proven to provide a mini-gut for understanding intestinal physiology (Sato et al., 2009). Therefore, to explore the effect of Ang4 on ISCs, we cultured organoids derived from small and large intestine with or without recombinant Ang4. Observation of organoids revealed that three indices for evaluating organoid growth, including organoid efficiency, budding, and area of cross section, were significantly increased when organoids were cultured with 5 μg/mL Ang4 (Figures 2A–D). To confirm the proliferation of IECs, we next performed immunofluorescence staining of organoids with Ki-67, which is well-established marker for cell proliferation. As expected, organoids cultured with Ang4 showed increased ratio of Ki-67^+^ cells indicating enhanced proliferation by Ang4 (Figures 2E,F). Lgr5+ ISC is a crucial cell subset, which can give rise to progenitor cells including absorptive enterocytes, secretory goblet cells, enteroendocrine cells, tuft cells, and Paneth cells (Beumer and Clevers, 2021). To evaluate the effect of Ang4 on Lgr5^+^ ISCs, we performed a qPCR analysis of the ISC signature genes in organoids cultured with or without Ang4. Following Ang4 stimulation for 4 days, the expression of ISC signature genes, including *Lgr5*, *Olfm4*, *Ascl2*, and *Smoc2*, significantly increased in small and large intestinal organoids when cultured with Ang4 (Figures 2G,H).

Since the crypt niche is comprised of IEC subsets, we compared the expression of gene sets defining distinct IEC subtypes between the control and Ang4-treated organoids. qPCR using cell-specific marker genes revealed that Paneth cell markers *Lyz1* and *Defa24* are markedly upregulated in Ang4-treated organoids, suggesting that Ang4 is indeed operative not only in Lgr5^+^ ISCs but also Paneth cells (Figure 2I). Taken together, these data indicate that Ang4 enhances organoid growth by increasing the expression of ISC signature genes and controlling crypt niche dynamics.

### 3.3 Ang4 activates Wnt and Notch signaling

**FIGURE 3:**
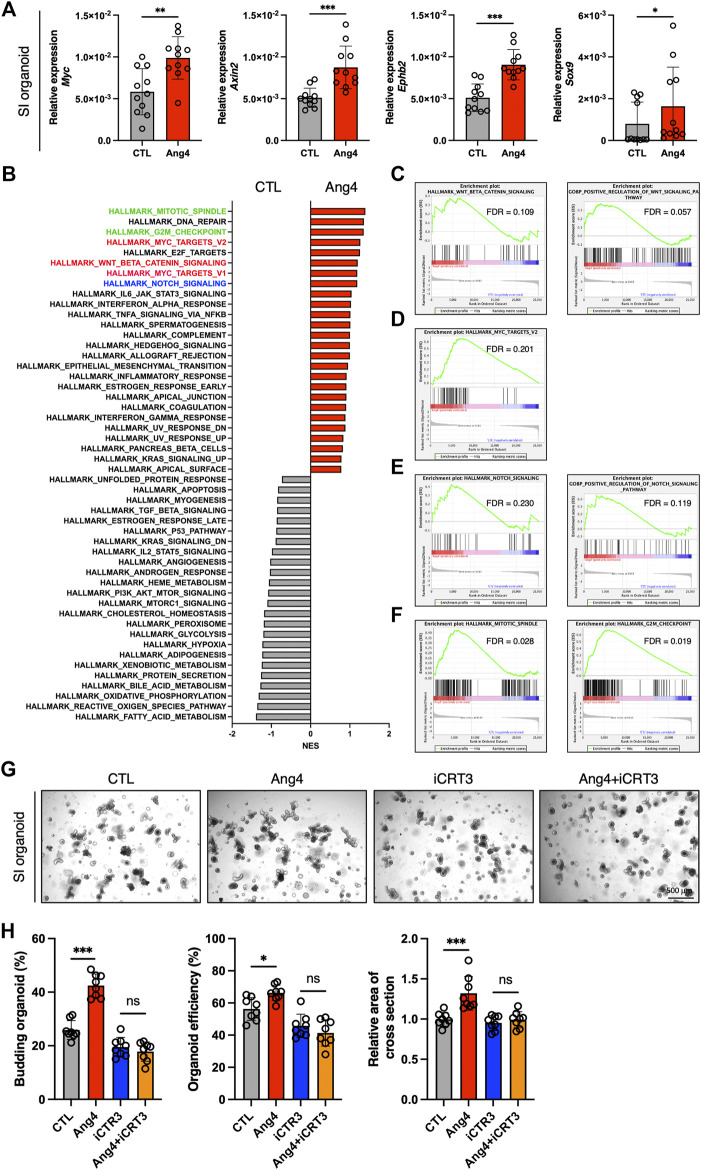
Ang4 activates Wnt and Notch signaling. **(A)** Expression of Wnt signaling target genes in organoids cultured with or without Ang4 at a concentration of 5 μg/mL (n = 11). **(B)** NES calculated by GSEA analysis with gene set of MSigDB’s hallmark collection. Gene sets highlighted by red, blue, and green indicated Wnt, Notch, and cell cycle related gene sets respectively. **(C)** Enrichment plot from gene set of Wnt beta catenin signaling. **(D)** Enrichment plot from gene set of Myc target. **(E)** Enrichment plot from gene set of Notch signaling. **(F)** Enrichment plot from gene set of mitotic spindle and G2M checkpoint. **(G)** Representative pictures of small intestinal organoids cultured with Ang4 and Wnt inhibitor iCRT3. **(H)** Area of a cross section, budding of organoids, and organoid efficiency shown in **(G)**. Data are pooled from two **(H, G)** or three **(A)** independent experiments and are presented as the mean ± SD; NS; not significant, *p < 0.05, **p < 0.01 via non-parametric Mann-Whitney test or ANOVA followed by Tukey’s multiple comparison test. RNA sequencing was performed by 4 samples in each group.

Wnt/β-catenin signaling plays an essential role in the maintenance and differentiation of Lgr5^+^ ISCs into terminal IEC subsets (Gehart and Clevers, 2019). For instance, IEC-specific deletion of T-cell factor 4, which is a Wnt effector molecule, leads to a significant loss of ISCs (van Es et al., 2012). In addition, conditional deletion of Wnt3 results in significantly reduces Lgr5^+^ ISCs and delays organoid growth, indicating that the Wnt ligand, Wnt3, is required for organoid growth and development (Farin et al., 2012). Based on these findings, we sought to determine whether Ang4 activates Wnt/β-catenin signaling. To assess the activation of Wnt/β-catenin signaling, we performed a qPCR analysis of Wnt target genes in organoids cultured with or without Ang4. When organoids were cultured with Ang4 for 4 days, we observed increased expression of well-established Wnt pathway target genes, including *Sox9*, *Axin2*, *Ephb2*, and *Myc* (Figure 3A). Next, we performed RNA sequencing and GSEA analysis for further analysis. Among 50 mouse hallmark gene sets, the gene sets related Wnt signaling and Myc target genes showed high normalized enrichment score (NES) when organoids were treated with Ang4 (Figure 3B). Enrichment plot of two gene sets revealed that Wnt pathway related genes and Myc target genes were significantly enriched in Ang4 treated organoids (Figures 3C,D). Furthermore, our GSEA analysis revealed the upregulation of Notch signaling in Ang4 treated organoids (Figures 3B,E). Hippo-Yap/Taz signaling is also essential for maintaining the stem cell pool and the balance between differentiation and proliferation in intestinal organoids (Gregorieff et al., 2015; Sancho et al., 2015). According to GSEA analysis, Hippo-Yap/Taz signaling related genes were not enriched in Ang4 treated organoids (Supplementary Figure S5A). Furthermore, we detected no change in the expression of the Hippo-Yap/Taz signaling target gene *Cyr61*, which is associated with intestinal organoid growth (Supplementary Figure S5B). In addition, we detected that cell proliferation-related genes, in gene sets of mitotic spindle and G2/M checkpoint, were highly enriched in Ang4 treated organoids (Figures 3B,F). This data was correlated with our observation that Ang4 enhanced organoid growth in Figure 2. Furthermore, RNA sequencing analysis revealed that Ang4 treatment increased its own expression (Supplementary Figure S6). This data suggests that Ang4 forms a positive feedback loop by increasing the number of Paneth cells that express Ang4, thereby promoting the growth of the organoids.

Next, we further investigated the association of Wnt signaling to Ang4 induced organoid proliferation using Wnt signaling inhibitor iCRT3 (Gonsalves et al., 2011). When the organoids were treated with Ang4 for 4 days, we detected a significant increase in three indicators including budding organoid number, organoid efficiency, and the area of cross-section. On the other hand, iCRT3 treated organoids didn’t respond to Ang4 stimulation, as evidenced by no difference in three parameters (Figures 3G,H). These data indicate that Wnt signaling is associated with the enhanced organoid growth induced by Ang4. Collectively, these data indicate that Ang4 activates Wnt/β-catenin and Notch signaling in intestinal organoids.

### 3.4 Ang4 increases ISCs and induces the proliferation of IECs *in vivo*

**FIGURE 4:**
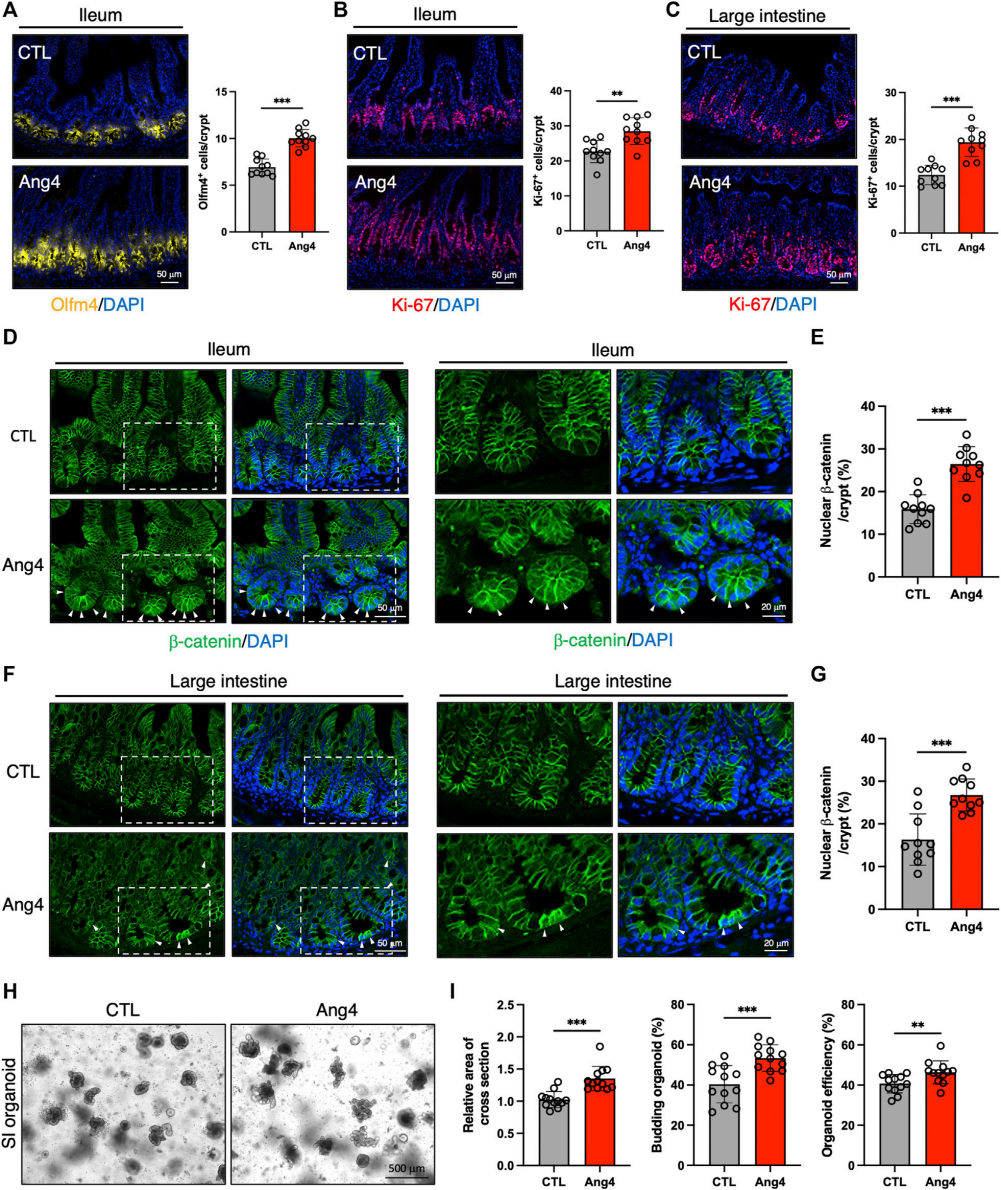
Ang4 induces expansion of ISCs *in vivo*. **(A)** Representative images of Olfm4 staining in ileal sections from Ang4 treated mice and quantification of Olfm4^+^ cells (n = 10). **(B)** Representative images of Ki-67 staining in ileal sections from control and Ang4 treated mice and quantification of Ki-67^+^ cells (n = 10). **(C)** Representative images of Ki-67 staining in colon sections from control and Ang4 treated mice and quantification of Ki-67^+^ cells (n = 10). **(D)** Representative images of β-catenin staining in ileal sections from control and Ang4 treated mice. White arrows indicate nuclear translocated β-catenin. left panel; low magnification, right panel; high magnification. **(E)** Quantification of nuclear β-catenin shown in **(D)** (n = 10). **(F)** Representative images of β-catenin staining in colon sections from control and Ang4 treated mice. White arrows indicate nuclear translocated β-catenin. Left panel; low magnification, right panel; high magnification. **(G)** Quantification of nuclear β-catenin shown in **(F)** (n = 10). **(H)** Representative pictures of 1st generation of organoid derived from control and Ang4 treated mice. **(I)** Area of a cross section, budding of organoids, and organoid efficiency shown in **(H)** (n = 12). Data are pooled from two **(H, I)** or three **(A–G)** independent experiments and are presented as the mean ± SD; **p < 0.01, ***p < 0.001 via non-parametric Mann-Whitney test.

Our *in vitro* findings indicated that Ang4 promotes the expression of ISC signature genes and enhances the growth of IECs. To validate the results from the *in vitro* organoid culture, we explored the effect of Ang4 *in vivo* by treating wild-type mice with recombinant Ang4. To address this, we administered Ang4 intraperitoneally on Days 0, 2, 4, and 6, and analyzed the results on Day 7. As expected, ileal sections from Ang4-administered mice displayed an increased number of ISCs expressing Olfm4, which is a well-established marker of ISCs (Figure 4A) (van der Flier et al., 2009). In addition, we detected an increased number of Ki-67^+^ cells in the TA zone in both the ileum and large intestines treated with Ang4 (Figures 4B,C). Furthermore, we also confirmed the translocation of β-catenin after Ang4 treatment. β-catenin translocates to the nucleus following the activation of Wnt signaling (Staal et al., 2008). Consistent with the upregulation of the Wnt target genes *in vitro*, enhanced translocation of β-catenin to the nucleus was observed in the small and large intestine of Ang4 treated mice (Figures 4D–G).

Next, we further confirmed the stemness by analyzing organoid forming efficiency with 1st generation of organoids derived from Ang4 treated mice. After 4 days from spreading freshly isolated crypts, organoids derived from Ang4 treated mice exhibited a higher area of a cross section, budding, and organoid efficiency compared to organoids from the control mice (Figures 4H,I). Collectively, these all data indicate that Ang4 increases ISCs and induces the proliferation of crypt cells accompanied by the activation of Wnt signaling *in vivo*.

### 3.5 Ang4 induces apoptosis at high concentrations

**FIGURE 5:**
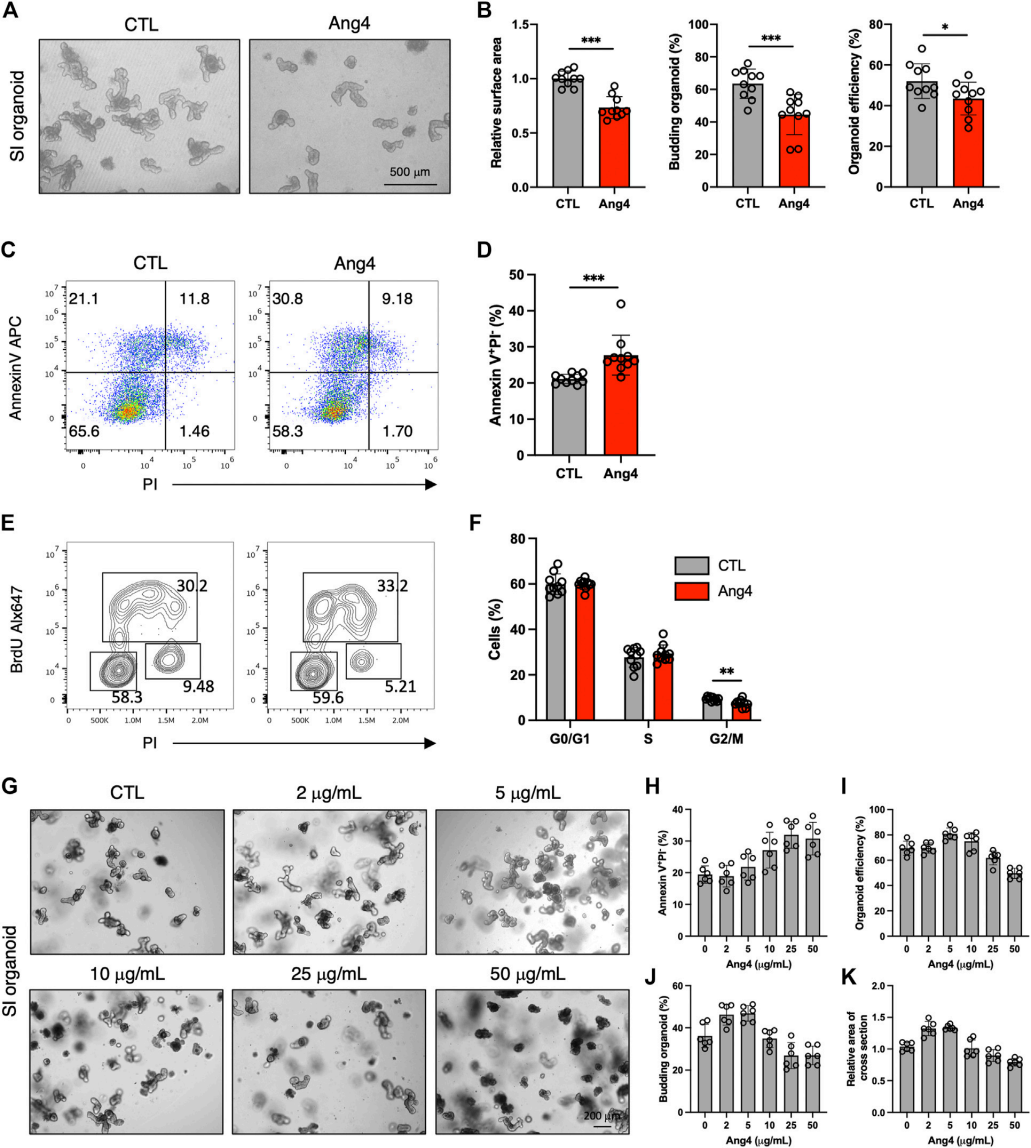
Ang4 suppresses cell proliferation and induces apoptosis at high concentration. **(A)** Organoids cultured with or without Ang4 at a concentration of 25 μg/mL. **(B)** Organoid efficiency, area of cross section, and budding organoids shown in **(A)** (n = 10). **(C)** Analysis of apoptosis in organoids cultured with or without Ang4 at a concentration of 25 μg/mL (n = 10). **(D)** The frequency of pre-apoptotic cells (Annexin V^+^PI^−^) shown in **(C)**. **(E)** Cell cycle analysis of small intestinal organoids cultured with or without Ang4 at a concentration of 25 μg/mL. **(F)** Frequency of cell cycle shown in **(E)** (n = 10). **(G)** Representative pictures of organoids cultured with or without Ang4 at a concentration of 2 μg/mL to 50 μg/mL (n = 6). **(H)** Frequency of apoptotic cells (Annexin V^+^PI^−^) analyzed by flowcytometry after treatment with indicated concentration of Ang4 (n = 6). **(I)** Organoid efficiency shown in **(G)** (n = 6). **(J)** Budding organoid shown in **(G)** (n = 6). **(K)** area of a cross section shown in **(G)** (n = 6). Data are pooled from two **(G–K)** or three **(A–F)** independent experiments and presented as the mean ± SD; *p < 0.05, **p < 0.01, ***p < 0.001 via non-parametric Mann-Whitney test.

AMPs protect against microbial infections and epithelial injury. However, a previous report revealed that AMPs exert side effects in mammalian cells in a context-dependent manner. For example, defensin induces tumor cell death by interacting with cell surface phosphatidylserine (Deslouches and Di, 2017; Parvy et al., 2019). Additionally, a previous study demonstrated that AMPs can induce apoptosis in mammalian cell lines in a dose-dependent manner (Paredes-Gamero et al., 2012). Based on these findings, we examined whether Ang4 induced apoptosis at high concentrations. We first treated the MC38 epithelial cell line, which was established from colon carcinoma, with Ang4 at concentrations ranging from 1 to 25 μg/mL for 24 h. Cell cycle analysis using BrdU and PI revealed that Ang4 significantly decreased the number of G2/M phase cells at a concentration of 25 μg/mL (Supplementary Figures S7A, B). Using Annexin V, which is a well-established cell marker for pre-apoptosis, and propidium iodide (PI), we detected an increase in apoptotic cells (Annexin V^+^PI^−^) after treatment with Ang4 at a concentration of 25 μg/mL (Supplementary Figures S7C, D). Next, organoids derived from the small intestine were treated with Ang4 at a concentration of 25 μg/mL for 4 days. As expected, the organoid efficiency, area of cross section, and budding were all significantly decreased when organoids were treated with Ang4 (Figures 5A,B). Consistent with the cell line experiments described above, Ang4-treated organoids showed increased apoptosis (Figures 5C,D). In addition, cell cycle analysis using BrdU and PI revealed that Ang4 significantly decreased the proportion of G2/M phase cells in small intestinal organoids (Figures 5E,F).

We next investigated whether induction of apoptosis byAng4 is specific. To assess this, we treat small intestinal organoids with 30 μg/mL of Reg3γ, which is an antimicrobial peptide expressed in intestinal tissues (Vaishnava et al., 2011). After 4 days of treatment, 3 indicates including budding organoid, organoid efficiency, and area of cross section showed no difference between control and Reg3γ treated organoids (Supplementary Figures S8A, B). In addition, while Ang4 treatment led to the increase of apoptotic cells (Annexin V^+^PI^−^), Reg3γ treatment showed no difference (Supplementary Figure S8C). These data suggest that the apoptosis induced by Ang4 might be specific at high concentrations.

We also examined the effect of Ang4 on ISCs at high concentrations. At 25 μg/mL, Ang4 upregulated ISC signature genes, similar to its effect at low concentrations (Supplementary Figure S9). We also investigated whether low concentrations of Ang4 induce apoptosis. At a concentration of 5 μg/mL, Ang4 did not affect apoptosis in organoids (Supplementary Figures 10A, B). Taken together, these findings indicate that Ang4 enhances ISC expansion but also suppresses IEC proliferation by inducing apoptosis in a context-dependent manner, particularly at high concentrations.

Our data indicate that the dose of Ang4 is critical for the balance of proliferation and apoptosis. Therefore, we further confirmed the kinetics of proliferation and apoptosis by treating small intestinal organoids with Ang4. Organoids were treated with Ang4 at the concentration of 0, 1, 5, 10, 25, and 50 μg/mL and monitored apoptosis by flowcytometry and measured organoid growth (Figure 5G). After treatment with Ang4 for 4 days, apoptosis defined as Annexin V^+^PI^−^ remained similar to the control at low concentrations ranging from 2 μg/mL to 5 μg/mL, but increased at concentrations from 10 μg/mL to 25 μg/mL. At a concentration of 50 μg/mL, the level of apoptosis was similar to that observed at 25 μg/mL (Figure 5H). Regarding organoid growth, the highest growth rate was observed at a concentration of 5 μg/mL evidenced by increased organoid efficiency (Figure 5I), budding (Figure 5J), and area of a cross section (Figure 5K). However, at concentrations higher than 10 μg/mL, organoid growth was decreased in a concentration-dependent manner (Figures 5I–K). These data indicate that proliferation and apoptosis induced by Ang4 are highly dependent on the dose *in vitro*.

**FIGURE 6:**
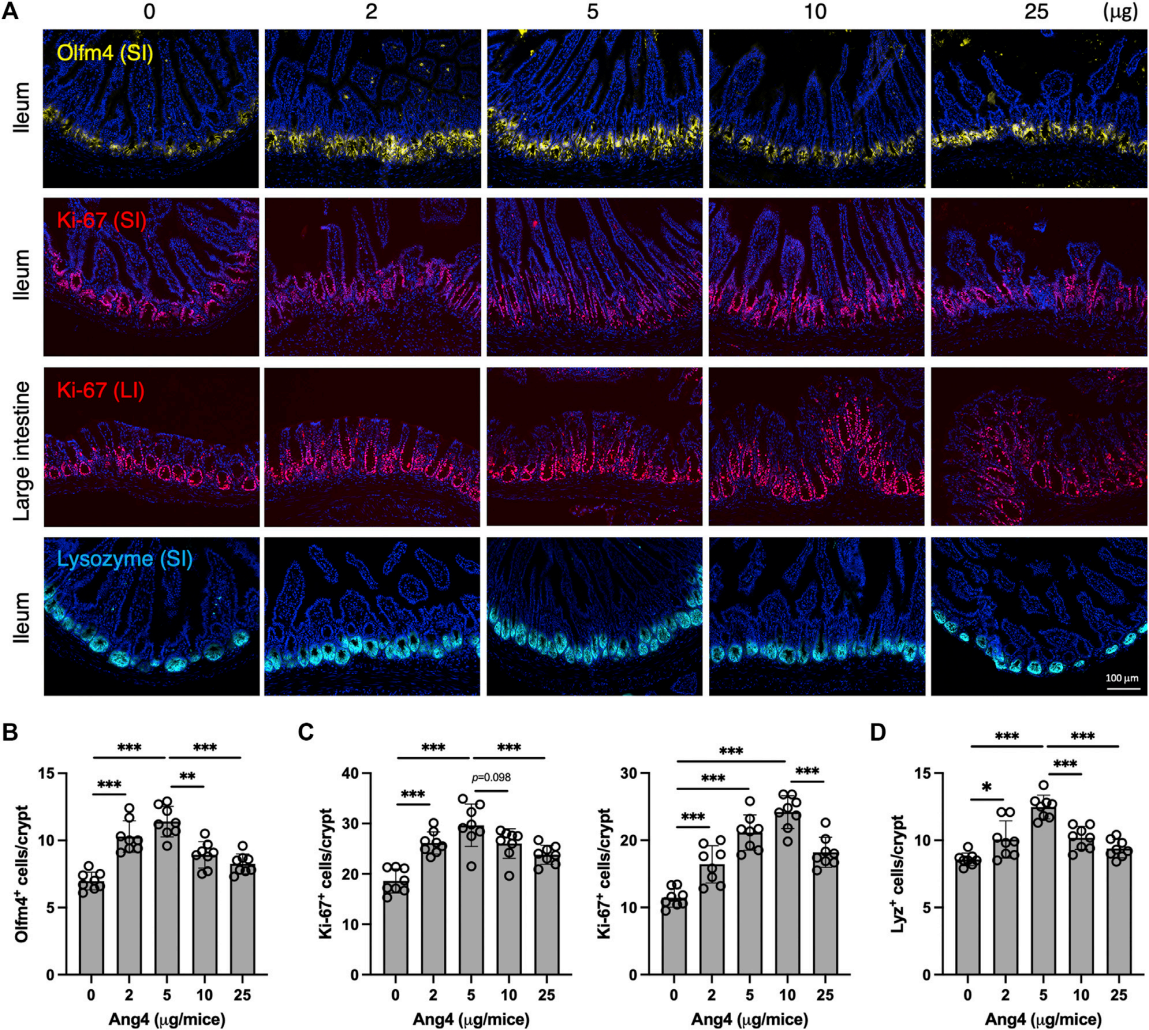
Concentration dependent effects of Ang4 *in vivo*. **(A)** Immunofluorescent staining of intestinal tissues from mice injected indicated amount of Ang4. **(B)** Quantification of Olfm4^+^ cells in ileum from An4 treated mice (n = 8). **(C)** Quantification of Ki-67^+^ cells in ileum (left panel) and large intestine (right panel) from An4 treated mice (n = 8). **(D)** Quantification of Lysozyme^+^ cells in ileum from An4 treated mice (n = 8). Data are pooled from two independent experiments and presented as the mean ± SD; *p < 0.05, **p < 0.01, ***p < 0.001 via ANOVA followed by Tukey’s multiple comparison test.

To further investigate our findings *in vivo*, we administrated Ang4 at doses ranging from 2 μg to 25 μg via i.p. injection every 2 days for a total of four injections. At day 7 from the initial Ang4 treatment, intestinal tissues were collected and stained with Olfm4, Ki-67, and Lysozyme respectively. We observed that the number of stem cells in the ileum, identified by Olfm4 in the small intestine, was increased at Ang4 dosing ranging from 2 μg to 5 μg (Figures 6A,B). On the other hand, 10 μg and 25 μg injections reduced stem cells compared to 5 μg administration with dose dependent. In addition, cell proliferation, marked by Ki-67, in the ileum and large intestines was highest at 5 μg and 10 μg, respectively, and subsequently decreased at the higher doses of Ang4 (Figures 6A,C). Furthermore, injection of 5 μg and 10 μg of Ang4 increased Paneth cells identified by Lysozyme, whereas 10 μg and 25 μg of Ang4 decreased Paneth cells in the ileum (Figure 6D). Consistent with these data, we detected an increase in low dose of Ang4 (2 μg/mL and 5 μg/mL) and a decrease in high dose Ang4 (10 μg/mL and 25 μg/mL) (Supplementary Figure 11). Collectively, these findings suggest that Ang4 exhibited the ability to induce proliferation and apoptosis in a concentration-dependent manner *in vivo*.

## 4 Discussion

Previous reports revealed that Paneth cells and goblet cells express Ang4 in mouse intestinal tissues. Recent transcriptomic technology has allowed the analysis of gene expression at single-cell resolution. Using single-cell RNA sequencing data from a public database, we demonstrated that Paneth cells express higher levels of Ang4 compared to goblet cells in the small intestine. Paneth cells secrete antimicrobial factors and contribute to Lgr5^+^ ISCs homeostasis through the expression of Wnt and Notch ligands (Sato et al., 2011; Cray et al., 2021). Similar to these soluble proteins, we propose Ang4 as a novel factor for maintaining ISCs in the crypt niche. In the presence of Ang4, organoids showed enhanced growth, and the mouse small intestine showed an increased number of ISCs. Furthermore, Ang4 is co-expressed with Reg4^+^ DCS cells, especially in the colon, which lacks Paneth cells. Reg4^+^ DCS cells express a wide range of soluble mediators, including Notch and Egf signaling and ablation of Reg4^+^ DCS cells leads to significant loss of Lgr5^+^ stem cells (Sasaki et al., 2016). We propose that Ang4-mediated maintenance of ISCs is one of the mechanisms by which Reg4^+^ DCS cells maintain Lgr5^+^ ISCs. Collectively, our *in vivo* and *in vitro* data strongly suggest a novel function of Ang4 in the maintenance of the crypt niche.

Although the Ang4 receptor has not been identified, the ability of Ang4 to induce Lgr5^+^ ISCs and organoid proliferation via activation of Wnt signaling is somewhat similar to the effects of R-spondin-1 in intestinal organoid cultures (Sato et al., 2009). R-spondin-1 is a potent Wnt signaling enhancer that is a strong agonist of LGRs and functions to maintain a high capacity for Lgr5^+^ ISC self-renewal (Koo and Clevers, 2014). Similarly, our data demonstrated that Ang4 enhances Lgr5^+^ stem cells in organoids accompanied by Wnt signaling activation. This effect may not be limited to the steady state and may extend to a mouse model of IEC injury, including experimental colitis and graft-versus-host disease. Furthermore, two major types of intestinal stem cells have been reported to maintain epithelial homeostasis (Yan et al., 2012). These two subsets are defined as Lgr5 marked fast-cycling crypt base columnar cells (CBC) and slow-cycling + 4 quiescent stem cell expressing *Hopx*, *Bmi1*, *Lrig1*, and *Tert* (Sangiorgi and Capecchi, 2008; Li and Clevers, 2010; Takeda et al., 2011). After treatment of small intestinal organoids with Ang4, we found a minor increase in *Hopx* expression, and no changes were observed in the expression of other +4 quiescent cell markers (Supplementary Figure S12). These data suggest that Ang4 does not affect on + 4 quiescent cells but rather exerts its effects on rapidly cycling stem cells that express Lgr5. These findings could provide insights into the detailed mechanism of how Ang4 acts on intestinal stem cells.

In mammals, the intestinal epithelium acts as the first line of defense against bacterial invasion. Therefore, Ang4 produced by IECs has been investigated in the epithelial cell defense system. However, we found unfavorable effects of Ang4 that high concentrations of Ang4 increase IEC apoptosis. A possible mechanism is the amphipathic structural feature of Ang4, which facilitates the binding of this protein with intestinal epithelial cells through electrostatic interactions, creating pores and ultimately leading to cell death or apoptosis (Zasloff, 2002; Paredes-Gamero et al., 2012). Through experiments using Reg3γ, it was suggested that the apoptosis induction by Ang4 might be specific. Moreover, plexinb2 is a functional receptor of human angiogenin (ANG) and is highly expressed in IECs, giving rise to the possibility of the interaction of human ANG with the plexin b2 receptor for internalization into epithelial cells (Yu et al., 2017; Bai et al., 2020). Although Ang4 specific receptors have not been identified, it is hypothesized that Ang4 may induce apoptosis by interacting with the Ang4-specific transmembrane receptor, transversing the plasma membrane, and entering the cytoplasm to disrupt mitochondrial integrity, which acts as a trigger for apoptosis (Barlow et al., 2006).

The significance of dual function of Ang4, inducing Lgr5^+^ stem cells and apoptosis, has to be discussed. Under the bacterial infections, such as *Salmonella*, Ang4 expression in the small intestine is upregulated rapidly, thereby the physiological concentration of Ang4 increases (Hooper et al., 2003; Walker et al., 2013). In the initial phase of the infection, Ang4 eliminates pathogens and induces IEC proliferation to enhance barrier function. After clearance of bacteria, Ang4 may inhibit epithelial proliferation by inducing apoptosis since excessive proliferation from ISCs such as hyperproliferation causes tumorigenesis of IECs (IJssennagger et al., 2012; Ijssennagger et al., 2015). In general, differentiation and proliferation processes of IECs are well controlled in both of steady state and inflammatory state (Allaire et al., 2018). Therefore, induction of apoptosis can be considered as a negative effect of Ang4, however it may facilitate homeostasis of intestinal tissues. Collectively, our study suggests that Ang4 orchestrates IEC differentiation and proliferation through its dual functions.

Comparing humans and mice studies is necessary to obtain a deeper insight and develop translational applications. Human angiogenin has only one gene, ANG, which is a homolog of mouse Ang4. The three-dimensional structures of human ANG and mouse Ang4, comprising α-helices and β-strands connected by loop structures, are remarkably similar. Protein sequencing analysis revealed 60.8% similarity in the amino acid sequence of human ANG and mouse Ang4. However, the functional sites of Ang4 occupy positions similar to their human ANG counterparts and are hypothesized to exhibit comparable activities (Iyer et al., 2013). For example, the catalytic sites responsible for RNase activity, nuclear localization sequence, and cell-binding segment are similar in human ANG and mouse Ang4 (Sheng and Xu, 2016). In addition, similar physiological functions, including angiogenesis, tumorigenesis, neuroprotection, antibacterial effects, and innate immunity, have been observed between mouse Ang4 and human ANG (Hooper et al., 2003; Sheng and Xu, 2016). Recent studies have demonstrated that human ANG may act as a diagnostic biomarker for several diseases such as cardiovascular diseases, cancer, and inflammatory bowel diseases (Yu et al., 2018). Therefore, regarding the structural and functional similarity between mouse Ang4 and human ANG, our findings strongly suggest that detailed studies of mouse Ang4 may lead to therapeutic targets for intestinal diseases, and research involving mouse Ang4 protein provides novel insights that could facilitate the study of human diseases.

Collectively, our study indicates that Ang4 has a dual function in IECs. First, Ang4 induces the expansion of Lgr5^+^ ISCs and promotes IEC proliferation. Second, Ang4 induces cell death in IECs, which is mediated by apoptosis at high concentrations. Since this dual function could be highly controlled in a dose-dependent manner, physiological concentrations and context-dependent expression of Ang4 need to be further studied. The premise that Ang4 exerts both beneficial and unfavorable effects may hold true for other AMPs. Overall, the significance of Ang4 as a crypt niche factor highlights its potential to facilitate the development of angiogenin-based treatment for intestinal diseases.

## Data Availability

All data are available in the main text and supplementary material. Raw illumina sequence data are available in the European Nucleotide Archive under accession number PRJEB62021.
