# Supplementary material for: Dual function of angiogenin-4 inducing intestinal stem cells and apoptosis
Source: Front Cell Dev Biol. 2023 Nov 2;11:1181145. doi: 10.3389/fcell.2023.1181145 (PMC10651741; doi:10.3389/fcell.2023.1181145)
Supplement: Supplementary file 1 [file DataSheet1.PDF]

Supplementary Material

Dual function of angiogenin-4 inducing intestinal stem cells and apoptosis

Hirohito Abo[1]*, Mst. Farzana Sultana[1,2], Hiroto Kawashima[1]*

*** Correspondence:**
Hirohito Abo, Ph.D.
habo@chiba-u.jp

Hiroto Kawashima, Ph.D.
h-kawashima@chiba-u.jp

Supplementary Figures

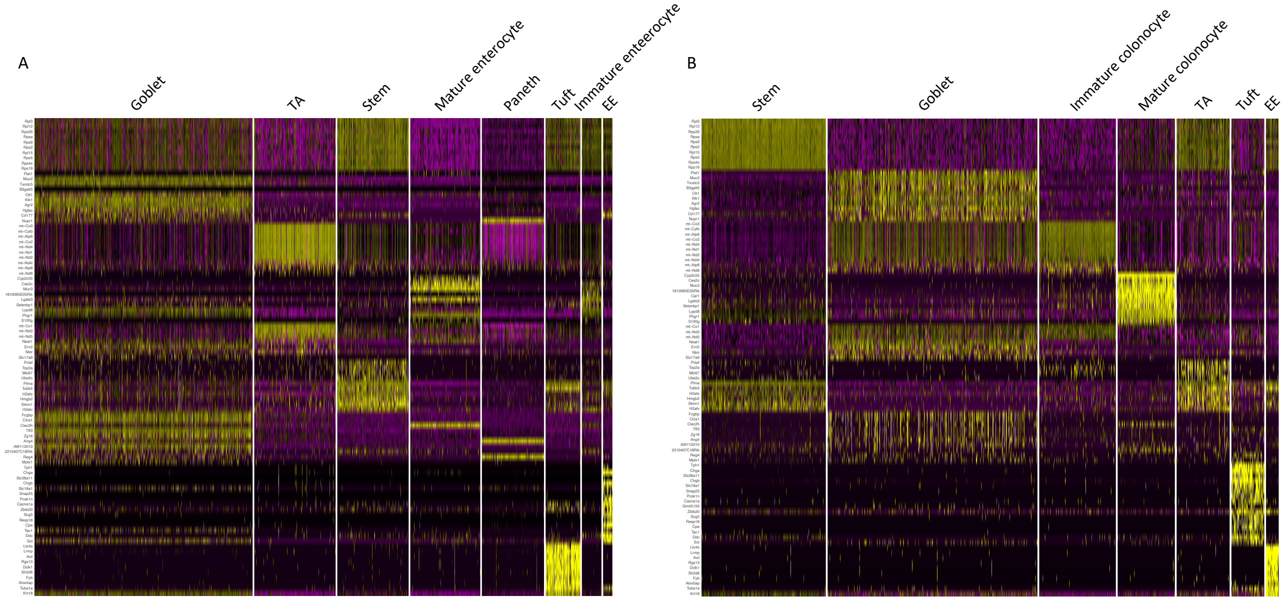

Supplementary Figure 1. Heatmap of defined IEC subsets.

(A) Heatmap of IEC subsets in small intestine. **(B)** Heatmap of IEC subsets in large intestine.

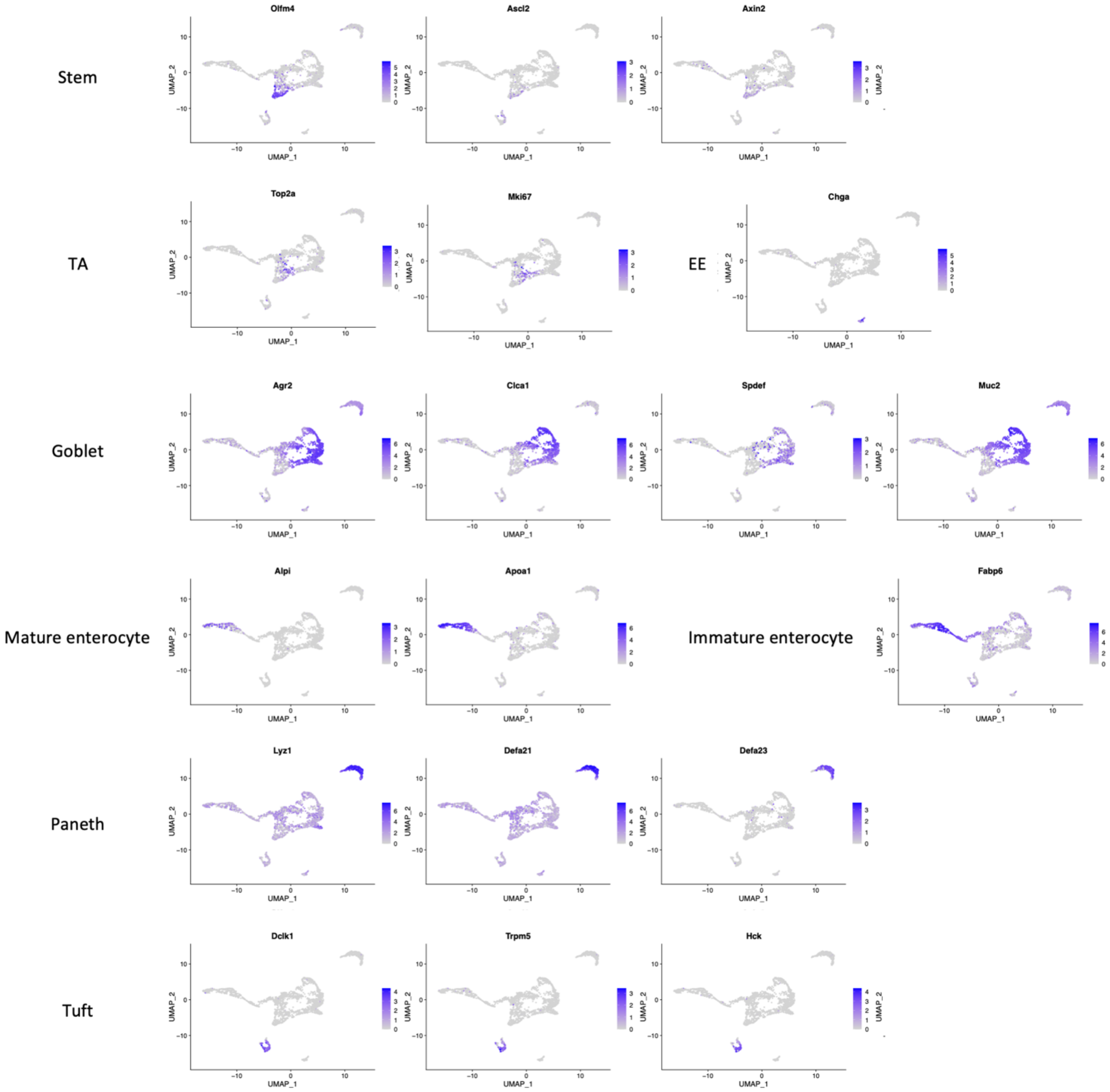

Supplementary Figure 2. Feature plot of genes used for IEC subset identification in small intestine.

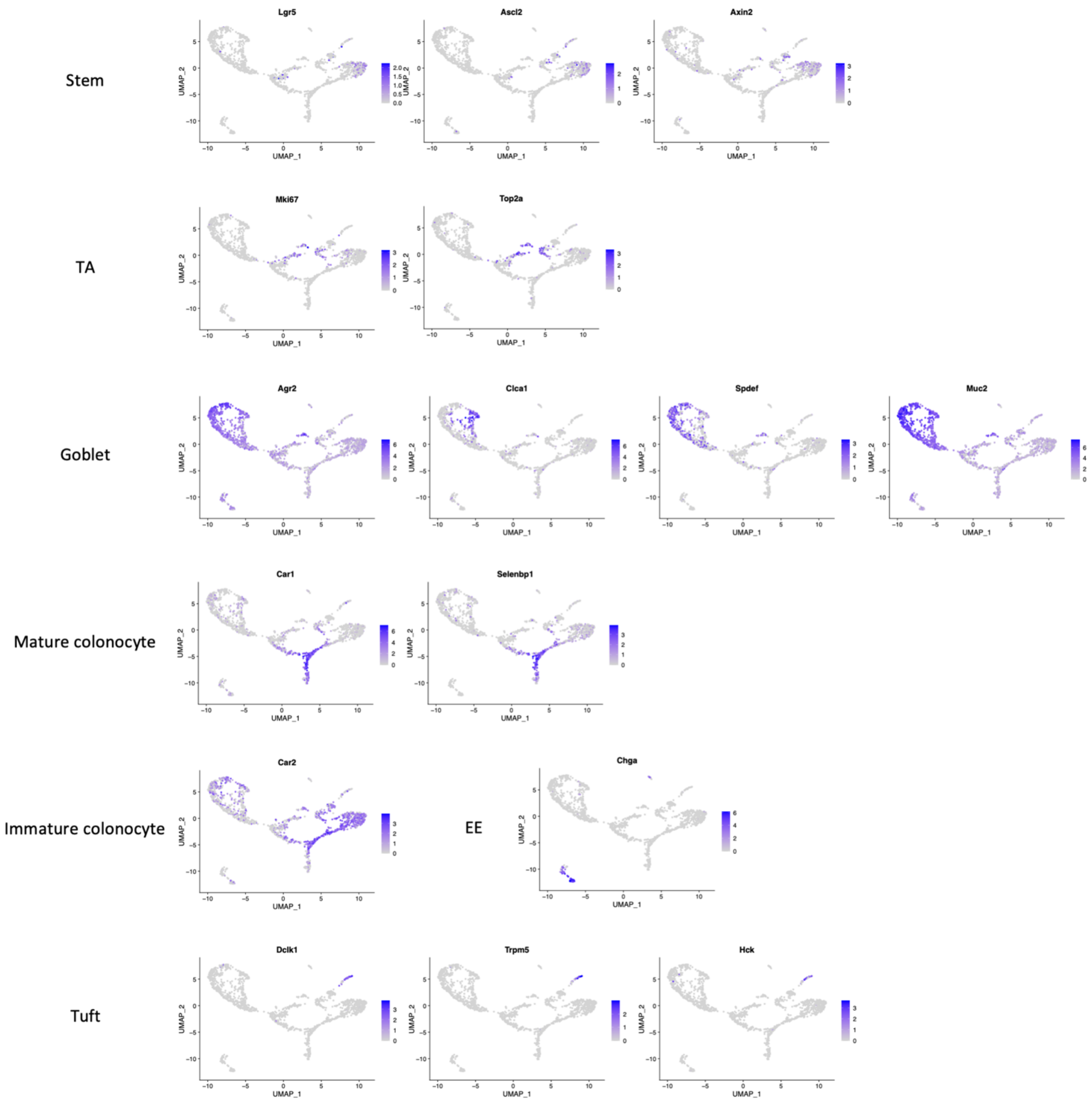

Supplementary Figure 3. Feature plot of genes used for IEC subset identification in large intestine.

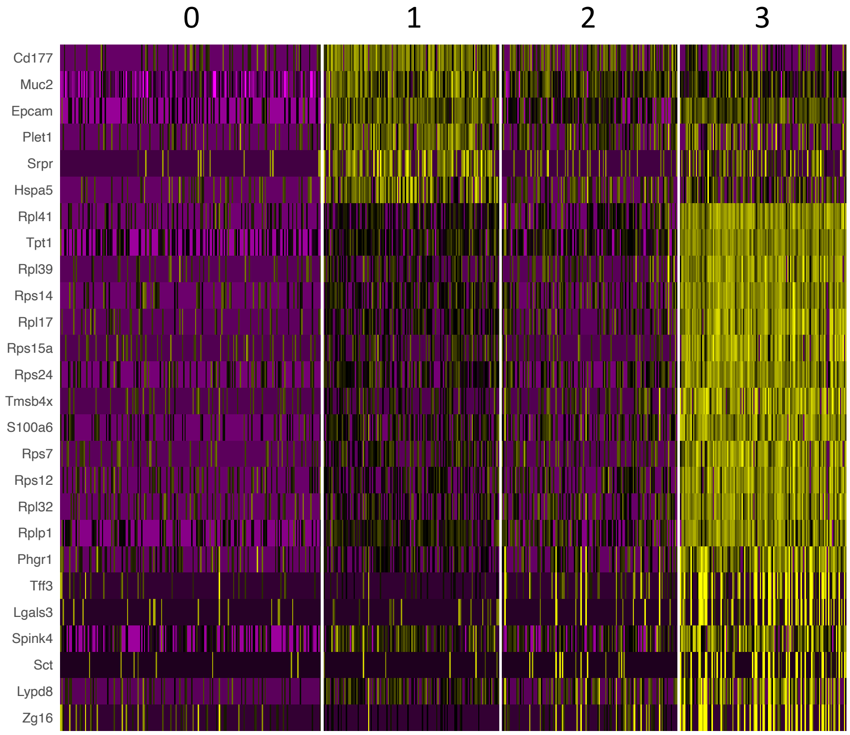

Supplementary Figure 4. Heatmap of subpopulations divided in four clusters on goblet cells.

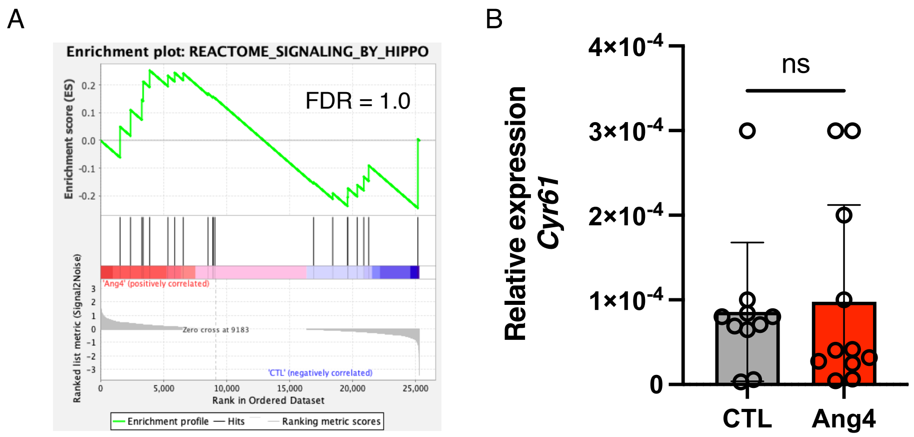

Supplementary Figure 5. Ang4 doesn't affect Hippo-Yap/Taz pathway.

(A) GSEA analysis using gene set of Hippo-Yap/Taz signaling. **(B)** qPCR analysis of Cyr61 (n=10-11). Data are pooled from three independent experiments and are presented as the mean ± SD; ns; not significant via non-parametric Mann-Whitney test. RNA sequencing was performed by 4 samples in each group.

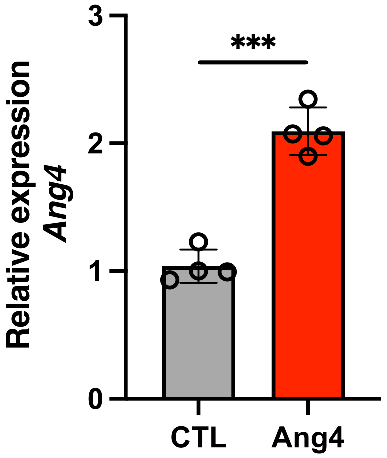

Supplementary Figure 6. Ang4 increases its own expression.

Expression of Ang4 on small intestinal organoid stimulated with Ang4 was analyzed by RNA sequencing. Data are presented as the mean ± SD; ****P* < 0.001 via non-parametric Mann-Whitney test. RNA sequencing was performed by 4 samples in each group.

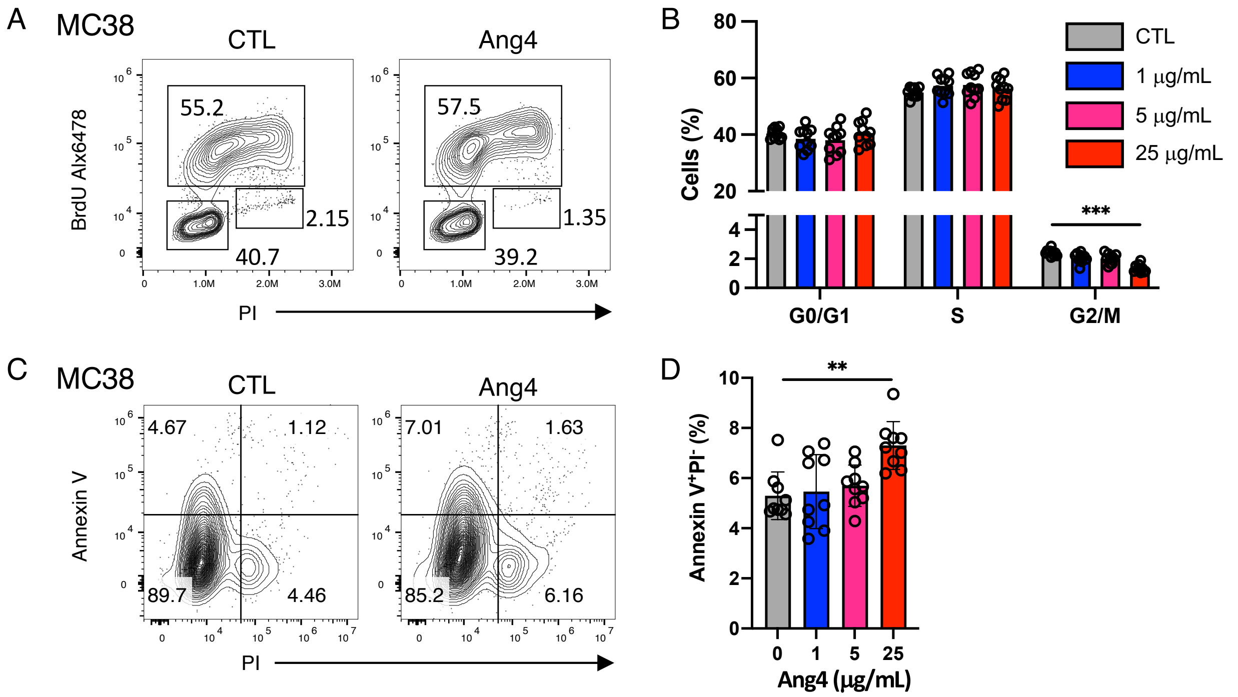

Supplementary Figure 7. Ang4 suppresses cell proliferation via inducing apoptosis on MC38 cell.

(A) Cell cycle analysis of the MC38 cell line cultured with or without Ang4 at a concentration of 25 μg/mL. **(B)** Frequency of cell cycle at a concentration of 0, 1, 5, and 25 μg/mL (n=9). **(C)** Analysis of apoptosis on MC38 cells treated with or without Ang4 at a concentration of 25 μg/mL. **(D)** Frequency of pre-apoptotic cells (Annexin $V^{+}PI^{-}$) at a concentration of 0, 1, 5, and 25 μg/mL (n=9). Data are pooled from three independent experiments and are presented as the mean ± SD; $**P < 0.01$, $***P < 0.001$ via ANOVA followed by Tukey's multiple comparison test.

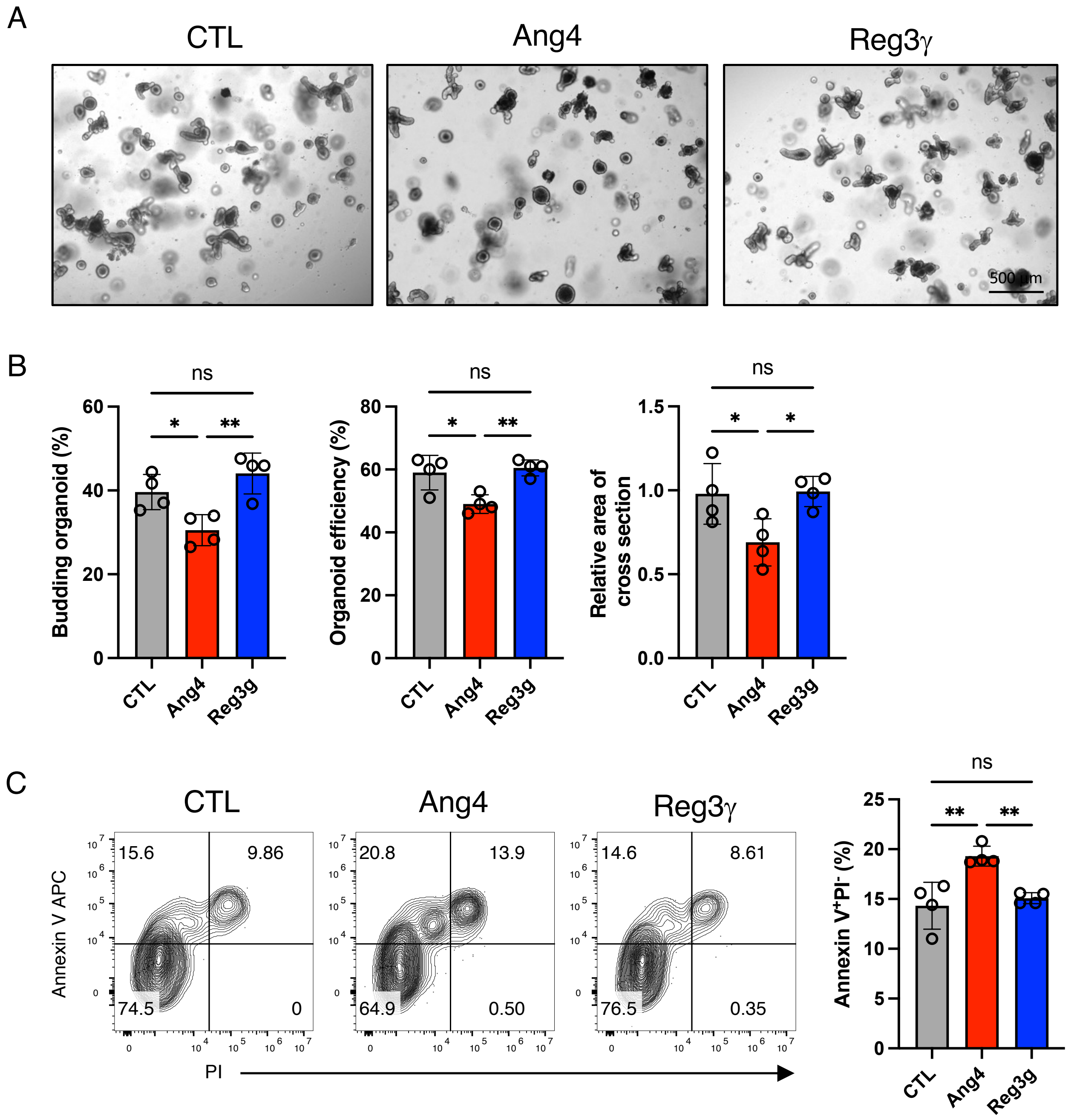

Supplementary Figure 8. Analysis of apoptosis in organoids treated with Ang4 and Reg3γ.

(A) Small intestinal organoids were treated with Ang4 and Reg3γ at concentration of 25 μg/mL and 30 μg/mL respectively. **(B)** Budding organoids, organoid efficiency, and area of cross section shown in (A) (n=4). **(C)** Analysis of apoptosis on organoids treated with Ang4 and Reg3γ (n=4). Data are presented as the mean ± SD; $^{**}P < 0.01$, $^{***}P < 0.001$ via ANOVA followed by Tukey's multiple comparison test.

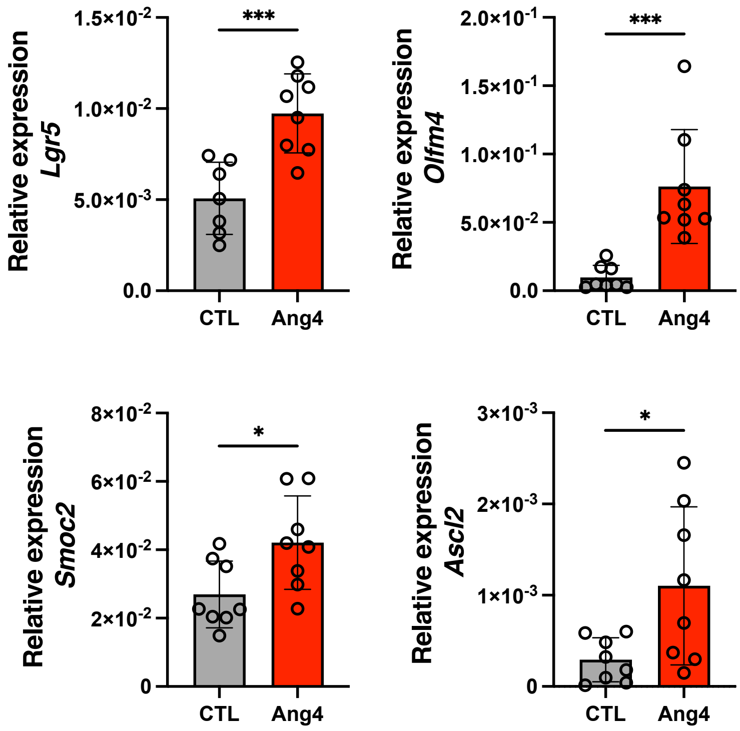

Supplementary Figure 9. Ang4 enhances the expression of ISC signature genes at high concentrations.

Expression of ISC signature genes in organoids cultured with Ang4 at a concentration of 25 μg/mL. Data are representative of two independent experiments and presented as the mean ± SD; *$P < 0.05$, ***$P < 0.001$ via non-parametric Mann-Whitney test.

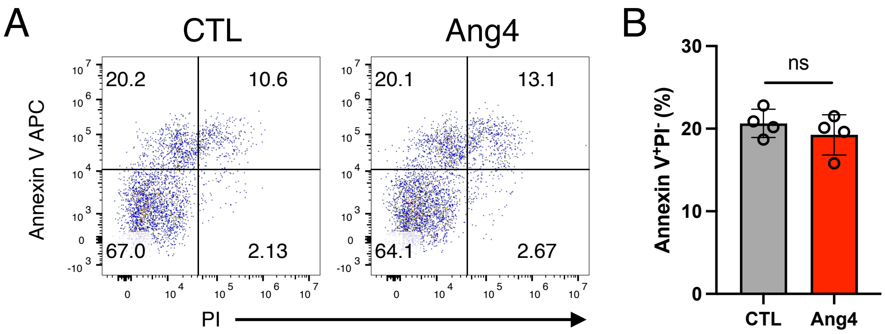

Supplementary Figure 10. Low concentration of Ang4 has no effect on apoptosis.

(A) Analysis of apoptosis on organoid cultured with Ang4 at 5 µg/mL. **(B)** Frequency of pre-apoptotic cells (Annexin V^+PI^-) shown in (A). Data are representative of two independent experiments and are presented as the mean ± SD; NS; not significant via non-parametric Mann-Whitney test.

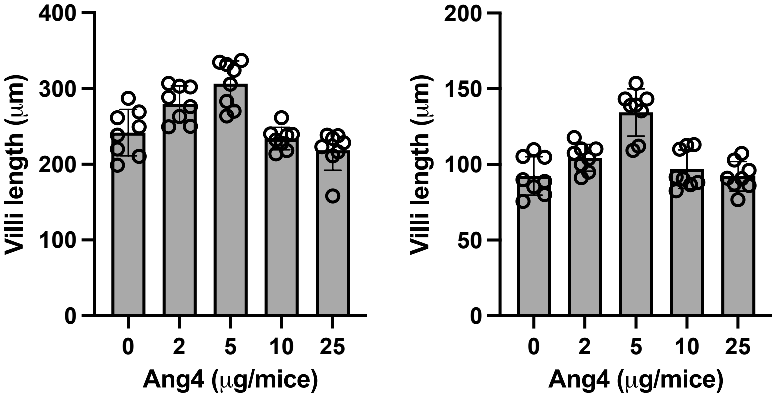

Supplementary Figure 11. Concentration dependent effects of Ang4 on the length of villi and crypt.

Villi and crypt length were measured in the mice treated with indicated amount of Ang4. Data are pooled from two independent experiments and presented as the mean ± SD.

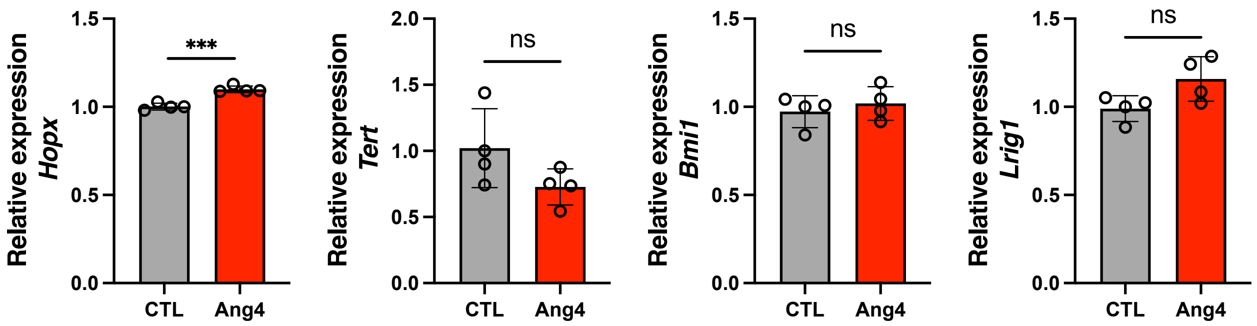

Supplementary Figure 12. Ang4 doesn't affect +4 quiescent stem cell.

Small intestinal organoids were stimulated with 5 μg/mL Ang4 and expression of +4 quiescent stem cell marker genes analyzed by RNA sequencing. Data are presented as the mean ± SD; ns; not significant, ***$P < 0.001$ via non-parametric Mann-Whitney test. RNA sequencing was performed by 4 samples in each group.
